# A Novel DFNA36 Mutation in *TMC1* Orthologous to the Beethoven (*Bth*) Mouse Associated with Autosomal Dominant Hearing Loss in a Chinese Family

**DOI:** 10.1371/journal.pone.0097064

**Published:** 2014-05-14

**Authors:** Yali Zhao, Dayong Wang, Liang Zong, Feifan Zhao, Liping Guan, Peng Zhang, Wei Shi, Lan Lan, Hongyang Wang, Qian Li, Bing Han, Ling Yang, Xin Jin, Jian Wang, Jun Wang, Qiuju Wang

**Affiliations:** 1 Chinese PLA Institute of Otolaryngology, Chinese PLA General Hospital, Beijing, China; 2 Beijing Institute of Otorhinolaryngology, Beijing Tongren Hospital, Capital Medical University, Beijing, China; 3 BGI-Shenzhen, Shenzhen, China; 4 BGI-Tianjin, Tianjin, China; 5 School of Bioscience and Biotechnology, South China University of Technology, Guangzhou, China; Universitat Pompeu Fabra, Spain

## Abstract

Mutations in the transmembrane channel-like gene 1 (*TMC1*) can cause both DFNA36 and DFNB7/11 hearing loss. More than thirty DFNB7/11 mutations have been reported, but only three DFNA36 mutations were reported previously. In this study, we found a large Chinese family with 222 family members showing post-lingual, progressive sensorineural hearing loss which were consistent with DFNA36 hearing loss. Auditory brainstem response (ABR) test of the youngest patient showed a special result with nearly normal threshold but prolonged latency, decreased amplitude, and the abnormal waveform morphology. Exome sequencing of the proband found four candidate variants in known hearing loss genes. Sanger sequencing in all family members found a novel variant c.1253T>A (p.M418K) in *TMC1* at DFNA36 that co-segregated with the phenotype. This mutation in *TMC1* is orthologous to the mutation found in the hearing loss mouse model named *Bth* ten years ago. In another 51 Chinese autosomal dominant hearing loss families, we screened the segments containing the dominant mutations of *TMC1* and no functional variants were found. *TMC1* is expressed in the hair cells in inner ear. Given the already known roles of *TMC1* in the mechanotransduction in the cochlea and its expression in inner ear, our results may provide an interesting perspective into its function in inner ear.

## Introduction

Hearing loss is the most common sensory disorder affecting one in 1000 births [1] and the prevalence rises to 2.7 per 1000 by the age of four [2]. More than 60% of cases can be attributed to genetic causes and inherited across generations. Hereditary hearing loss is a highly heterogeneous disorder. So far, a total of 76 non-syndromic hearing loss genes have been identified, including 31 autosomal dominant, 47 autosomal recessive and four X-linked genes (http://hereditaryhearingloss.org). Among these genes, eight of them are inherited in both autosomal dominant and recessive patterns, such as *TMC1* (http://hereditaryhearingloss.org/).

*TMC1* was identified as a causative gene for both autosomal dominant (DFNA36) and autosomal recessive (DFNB7/11) non-syndromic hearing loss by Kurima and colleagues in 2002[3]. Based on their results, Vreugde and colleagues screened the *Tmc1* gene and found the p.M412K mutation in a hearing loss mouse model named *Bth* which was arisen in a large-scale ENU mutagenesis program [4], [5]. Since then, more than 30 autosomal recessive mutations in *TMC1* have been reported in DFNB7/11 families. *TMC1* is identified as a common gene associated with non-syndromic hearing loss with a frequency up to 6.6% in Turkey [3], [6]–[17]. In contrast, only two amino acid residues with three mutations have been reported to be associated with autosomal dominant hearing loss [3], [14], [18], [19]. One is the amino acid-572 with two mutations at this site, p.D572N and p.D572H. These two mutations have been observed in three unrelated North American families with non-syndromic, post-lingual, progressive sensorineural hearing loss [3], [18], [19]. The other residue is the amino acid-417 with mutation p.G417R [14]. This mutation is adjacent to the *Bth* mouse mutation in the *Tmc1* gene, which may have similar function consequences. However, the mutation orthologous to p.M412K in murine *Tmc1* has yet to been found in human hearing loss family since it has been reported in 2002 [5]. Here, in a large Chinese family (1304) of six-generation with autosomal dominant hereditary hearing loss, we identified a novel mutation of p.M418K in *TMC1* through sequencing the whole exome of the proband, which is important and beneficial to discover the pathologic mechanism of DFNA36 hearing loss caused by *TMC1* mutation.

## Methods

### Ethics Statement

The study was approved by the Committee of Medical Ethics of Chinese People's Liberation Army (PLA) General Hospital. All the samples were analyzed under the appropriate ethical approvals, and written informed consents were obtained from all subjects or caregivers. Next of kin, care takers or guardians consented on the behalf of minors/children participants whose capacity to consent was compromised.

### Family Recruitment and Clinical Evaluations

Family 1304 were ascertained from the Department of Otolaryngology, Head and Neck Surgery, at the Institute of Otolaryngology of PLA, Chinese PLA General Hospital. Members of this family were interviewed by a team of experienced ear, nose and throat doctors, and physicians to identify either personal or family medical evidence of hearing loss, tinnitus, vestibular symptoms, use of aminoglycosides, and other clinical abnormalities. Audiometric evaluations included pure tone audiometry using Madsen Orbiter 922 audiometer (Denmark), auditory brainstem responses (ABR) and distortion product otoacoustic emissions (DPOAE) using SmartEP of Intelligent Hearing system (USA). The audiological data were evaluated based on the criteria established by European Working Group on Genetics of Hearing loss [20]. High resolution computed tomography (HRCT) was also performed on some subjects to verify whether the family members had other complications in addition to hearing disorders.

A total of 51 unrelated autosomal dominant hearing loss families were chosen as the other affected set for further analysis. In these families, mutations in common genes associated with hearing loss, such as *GJB2*, *SLC26A4* and mitochondrial DNA A1555G, were all excluded. As a comparison group, 100 unaffected individuals of matched geographical ancestry were recruited in this study.

### DNA Sample and Exome capture

Peripheral blood samples were obtained and genomic DNA was extracted according to standard procedures. Qualified genomic DNA samples (6 ug) of the proband were sheared by sonication. Then the fragment of each shared genomic DNA sample was hybridized to the SureSelect Biotiny lated RNA Library (BAITS) for enrichment.

### NGS, reads mapping and SNPs detection

The enriched libraries were loaded on the HiSeq 2000 platform to be sequenced. Raw image files were processed by Illumina Pipeline v1.6 for base-calling with default parameters and the sequences of each individual were generated as 90 bp paired-end reads. Then the sequenced raw data was aligned to the NCBI human reference genome (NCBI 36.3) using SOAPaligner [21]. After that, the duplicated reads were filtered out and the clean reads located in the target region were collected. The consensus of genotype and quality were estimated by SOAPsnp (version1.03) using the clean reads. The variations of low quality were filtered out according to the following criteria: (i) quality score <20 (Q20); (ii) average copy number at the allele site > = 2; (iii) distance of two adjacent SNPs <5 bp; and (iv) sequencing depth <4 or >500.

### Detection of insertions and deletions

Insertions and deletions (indels) in the exome regions were identified through the sequencing reads. We aligned the reads to the reference genome by Burrows-Wheeler Aligner (BWA0.5.8) [22], and passed the alignment results to the Genome Analysis Toolkit (GATK1.0.4705) to identify the breakpoints. Finally, we annotated the genotypes of insertions and deletions [23].

### Sequencing analysis of candidate gene

Candidate variants located in previously reported hearing loss genes found in exome sequencing were screened in all available members from family 1304. Genotype of the variant c.1253T>A (p.M418K) in *TMC1* was found to be co-segregated with the hearing loss in family 1304. As the candidate gene, the variant in *TMC1* was screened in 100 unaffected individuals geographically matched. In addition, 51 unrelated autosomal dominant hearing loss families without *GJB2*, *SLC26A4* and mitochondrial DNA A1555G mutations were chosen as another affected set for further analysis. For this cohort, exon16, exon19 and their flank sequences containing all the four mutations associated with dominant hearing loss were sequenced applying Sanger sequencing. Primer pairs were designed using the online Primer 3.0 software and synthesized by Invitrogen (Table S1, Beijing, China) to amplify each exon and boundaries.

## Results

### Clinical description

**Figure 1:**
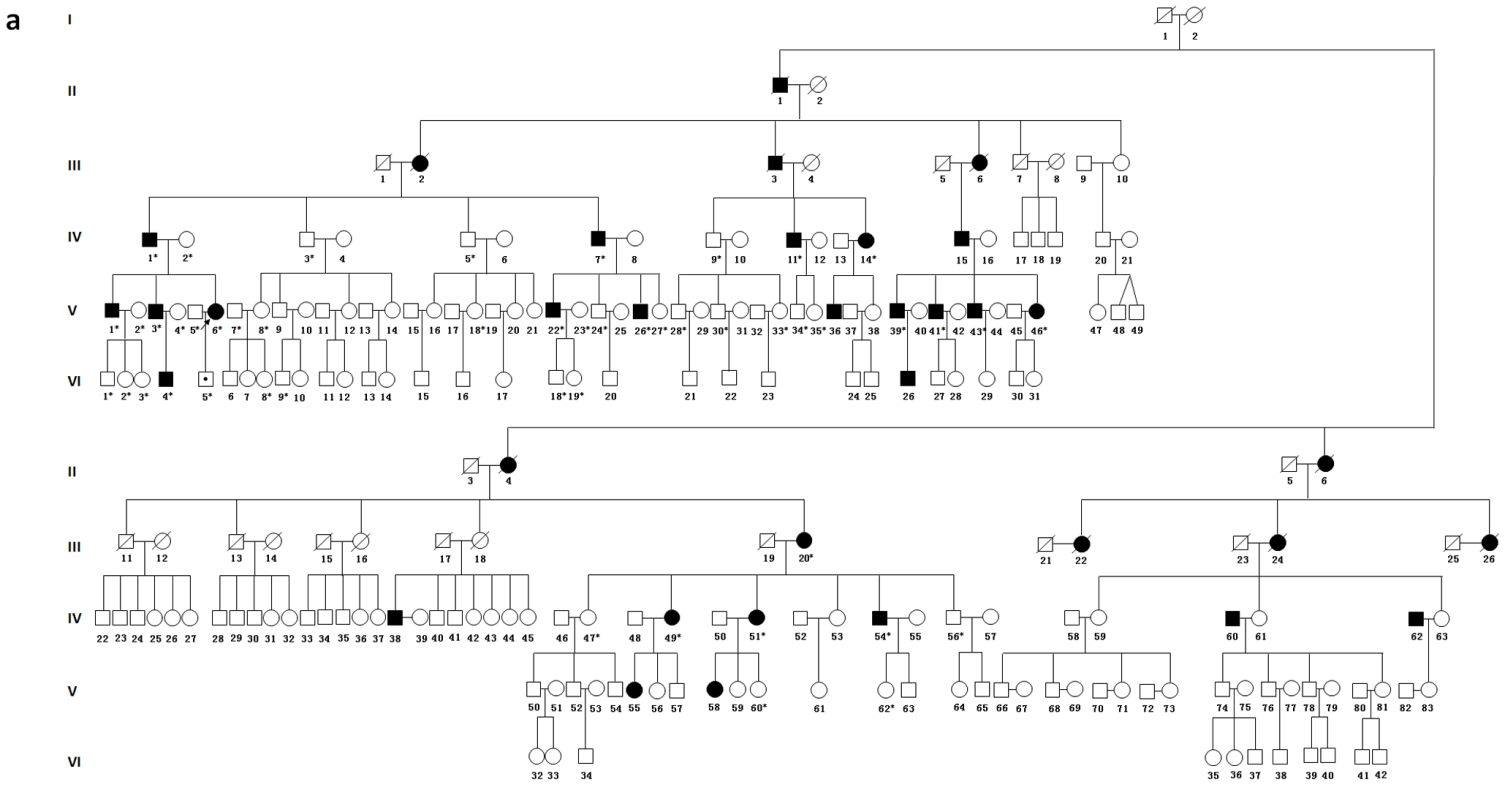
Pedigree of family 1304. Filled symbols for males (squares) and females (circles) represent affected individuals, and empty, unaffected ones. An arrow denotes the proband. A symbol with dot indicates the individual younger than the average age of onset, who is mutation carrier but does not present hearing loss (a mutation carrier). Symbols with asterisk are individuals who have had clinical and genetic tests.

**Figure 2:**
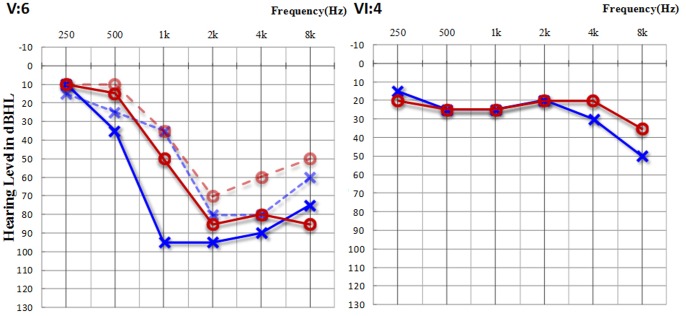
Audiograms of the proband (V:6) and the a five-year-old patient (VI:4). Symbols “o” and “x” denote air conduction pure-tone thresholds at different frequencies in the right and left ear. dB, decibels; Hz, Hertz. The dashed line represent the audiograms detected in 2005 when the proband (V:6) was 23 years old. Audiological examination with solid line was performed in 2012.

Family 1304 was a six-generation pedigree with 222 members, 35 of whom suffered hearing loss (Figure 1). This family was originated from Hebei province in North China. In this study, 48 members were under detailed physical examinations and audiometric evaluations (Figure 1), and 18 of them showed post-lingual, progressive, and symmetric sensorineural hearing loss with high frequency tinnitus (Figure 2, and Table 1). The onset age ranged from 5 to 28 years old. Hearing loss appeared to initially affect high frequencies with mild or moderate levels at the onset age and progressed to profound levels by the fifth or sixth decade. The low-frequency hearing deteriorated to profound levels and the audiometric graph changed to flat pattern eventually.

**Table 1:** Summary of the clinical data for all individuals with p.M418K in family 1304.

ID	Gender	Age of test (years)	Age of onset (years)	PTA	Severity of hearing loss	Tinnitus	Genotype
III:20	Female	70	NA	L:80.00	severe	L:durative	T/A
				R:83.75	severe	R:durative	
IV:1	Male	62	20	L:105.00	profound	L:durative	T/A
				R:107.50	profound	R:durative	
IV:7	Male	53	17	L:68.75	moderate	L:durative	T/A
				R:75.00	severe	R:durative	
IV:11	Male	44	24	L:83.75	severe	L:durative	T/A
				R:71.25	severe	R:no	
IV:14	Female	52	20	L:92.50	severe	L:no	T/A
				R:93.75	severe	R:no	
IV:49	Female	46	5	L:98.75	profound	L:durative	T/A
				R:101.25	profound	R:durative	
IV:51	Female	42	13	L:77.50	severe	L:durative	T/A
				R:81.25	severe	R:durative	
IV:54	Male	38	20	L:55.00	moderate	L:durative	T/A
				R:60.00	moderate	R:durative	
IV:60	Male	64	20	L:76.25	severe	L:durative	T/A
				R:77.50	severe	R:durative	
V:1	Male	37	18	L:80.60	severe	L:intermittence	T/A
				R:93.75	severe	R:intermittence	
V:3	Male	26	15	L:73.75	severe	L:durative	T/A
				R:71.25	severe	R:durative	
V:6	Female	23	13	L:55.00	moderate	L:durative	T/A
				R:43.75	moderate	R:durative	
V:22	Male	27	15	L:62.50	moderate	L:intermittence	T/A
				R:66.25	moderate	R:intermittence	
V:26	Male	20	15	L:62.50	moderate	L:intermittence	T/A
				R:66.25	moderate	R:intermittence	
V:39	Male	53	10	L:105.00	profound	L:durative	T/A
				R:105.00	profound	R:durative	
V:41	Male	45	28	L:73.75	severe	L:durative	T/A
				R:82.50	severe	R:durative	
V:43	Male	35	20	L:75.00	severe	L:durative	T/A
				R:71.25	severe	R:durative	
VI:4	Male	5	5	L:25.00	mild	L:no	T/A
				R:22.50	mild	R:no	
VI:5	Male	2	NA	L:NA	NA	NA	T/A
				R:NA	NA	NA	

PTA, Pure Tone Average. NA, Not Available. L, left ear; R, right ear; T/A in "Genotype" column indicates the genotype of the patient in the mutation of c.1253T>A (p.M418K).

**Figure 3:**
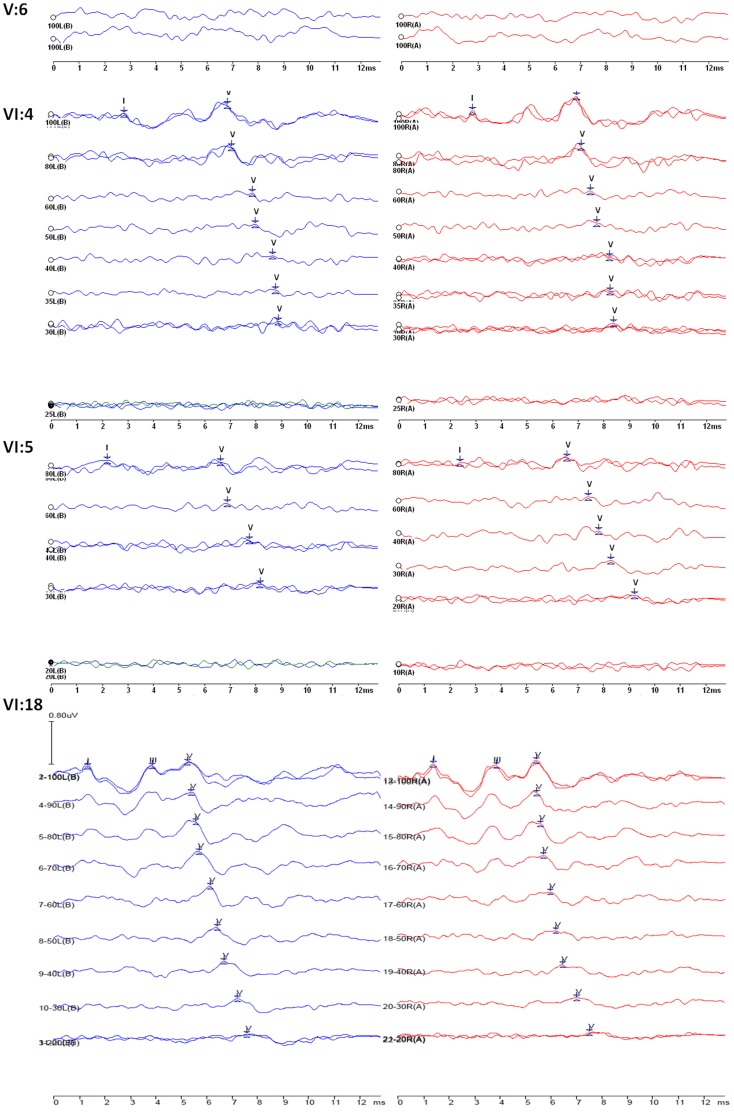
ABR results performed to the proband (V:6), a five-year-old patient (VI:4), a two-year-old carrier (VI:5) of p.M418K and a five-year-boy (VI:18) with normal hearing and wildtype genotype. ABR was performed using click stimulus.

The proband (V:6) was a 23-year-old female suffering from hearing loss accompanied by tinnitus when she first visit the outpatient clinic in the year of 2005. She showed a severe hearing loss at 2 kHz and 4 kHz and a moderate hearing loss at 1 kHz and 8 kHz. Seven years later, the threshold increased about 15–30 dB HL at 1 kHz–8 kHz in the right ear, 10–15 dB HL at 2 kHz–8 kHz in the left ear (Figure 2). The threshold of the left ear at 1 kHz increased 60 dB HL within seven years. ABR could not be evoked in both ears (Figure 3). DPOAEs were absent at all frequencies. The youngest patient of this family (VI:4) was 5 years old. And behavior audiometry to him showed bilaterally mild hearing loss (Figure 2). The threshold of ABR was 30 dB nHL. The latencies of wave I, III and V were prolonged and the I-to-V interwave latencies were normal in both ears. The waveform morphology was abnormal and the amplitudes were lower compared to normal waveform morphology (Figure 3). DPOAEs were present from 2.5 kHz to 8 kHz and other frequencies were absent in the left ear, while in the right ear, it was evoked from 2 kHz to 8 kHz and other frequencies were not elicited. High resolution CT scan showed normal middle and inner ear structure, including normal vestibular aqueduct and internal auditory canal.

### Exome sequencing

**Figure 4:**
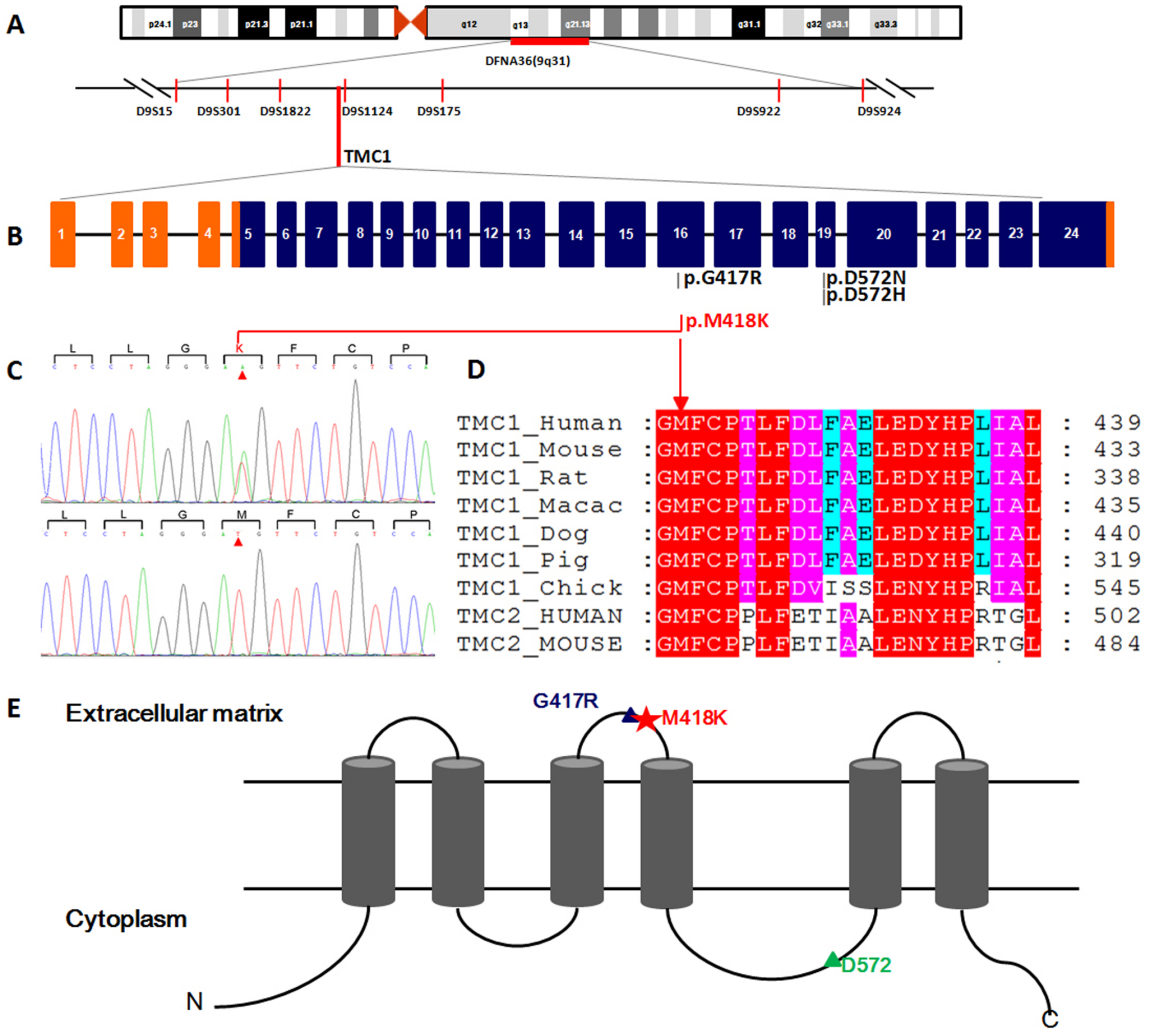
Gene identified in family 1304 with autosomal dominant progressive hearing loss. A: Schematic physical and genetic maps of DFNA36 locus on the 9q31chromosomal region. The *TMC1* gene is indicated. B: Schematic structure of *TMC1* gene. *TMC1* gene has 24 exons. Mutation of p.M418K and p.G417R locate in exon16, and mutation of p.D572N and p.D572H are in exon19. C: Sequencing chromatograms of *TMC1* showing the heterozygous substitution, c.1253A>T in affected individuals (upper panel) compared with that of normal control (lower panel). The mutated nucleotides are marked by triangles. The predicted amino acid changes and surrounding ones are indicated above the sequences. D: Multiple amino acid sequences alignment of *TMC1* and its paralog of *TMC2* using ClustalW software. The conservation analysis shows that p.M418K(arrow) mutation in *TMC1* located in a highly conserved position comparing with the corresponding sequences of human, mouse, rat, macaque, dog, pig, chick, human *TMC2* and mouse *Tmc2*. E: A schematic diagram of *TMC1* protein predicted by TMHMM2.0 containing six transmembrane domains, a cytoplasmic N and C termini. All reported DFNA36 mutations or residual are indicated. Mutation of p.M418K found in this study is located in the second extracellular loop between the third and the fourth transmembrane domain.

Whole exome of the proband was sequenced and an average of 4.7 billion bases of sequence were generated as paired-end, 90 bp reads, about 63.4% of the total bases were mapped to the targeted bases with a mean coverage of 53.7-fold. At this depth of coverage, 98.3% of the targeted regions were sufficiently covered to pass our thresholds for variant calling (Table S2, Figure S1 and Figure S2). A total of 38130 single nucleotide variants and 2220 indels were identified by exome sequencing (Table S3 and Table S4). Among these variants, we focused on non-synonymous (NS) variants, splice acceptor and donor site variants (SS), and short, frame-shift coding indels that were more likely to be pathogenic mutations than other variants. A total of 7477 variants following the above inclusion criteria were detected in the proband of family 1304 (Table 2). Hereditary hearing loss is a monogenic and always caused by rare variants, frequency of which may be rare or absent in the general population. Therefore, we compared all identified NS/SS/Indel variants in the proband against dbSNP132, the 1000 Human Genome Project (201003 released), eight previously exome-sequenced HapMap samples (HapMap 8), and YH SNPs (Table 2), and removed the shared SNPs. After this filter process, the candidate list reduced to 635 NS/SS/Indel variants. Four of the 635 NS/SS/Indel variants, p.M418K (*TMC1*), p. R382C (*ESRRB*), p.S385P (*ESPN*) and p.D2E (*WFS1*) located in the known hearing loss genes, which may attribute to the phenotypes of the family. Then these four variants were sequenced in all available members of family 1304 by Sanger sequencing. It was found that the mutation of c.1253T>A (p.M418K, NM_138691) in *TMC1* gene completely co-segregated with the phenotype and all 18 patients were heterozygous on this site (Figure 4A, 4B, 4C). In addition to the clinically diagnosed patients, there was a two-year-old boy (VI:5) who carried this mutation without hearing loss (Figure 1). This mutation was not detected in any of the 100 geographical matched controls. The other three candidate variants did not co-segregate with the members of family. To access the likelihood that whether the variant p.M418K in *TMC1* gene is functional or not, we used SIFT software (vision 4.0.3) to predict the biophysical consequences of this substitution and found that this variant is likely to be deleterious. Alignments of the amino acid sequences of *TMC1* in human species, mouse, rat, macaque, dog, pig and chick as well as with human *TMC2* and mouse *Tmc2* showed that the mutation is located in a highly conserved position, which is homologous to the p.M412K mutation in the *Bth* mouse inherited in autosomal dominant pattern (Figure 4D).

**Table 2:** Filter process for the variants found by whole exome sequencing.

Filter process	V:6
Functional_variations	7477
Against_dbSNP_132	1051
Against_dbSNP_1000 Genomes	723
Against_dbSNP_1000 Genomes _Hapmap 8	639
Against_dbSNP_1000 Genomes _Hapmap 8_YH	635
Variants in genes associated with hearing loss	4

The number of functional variants (non-synonymous/splice acceptor and donor site/insertions or deletions) is listed under various filters. Variants were filtered by presence in dbSNP, 1000 Genomes, HapMap 8 or YH.

To further analyze the contribution of *TMC1* for autosomal dominant hearing loss, we successfully screened the exon16 and exon19 in another 51 autosomal dominant hearing loss families. No functional variants were found to be associated with hearing loss in these families.

## Discussion

In this study, we identified a novel mutation p.M418K in *TMC1* in a Chinese family of six generation using the strategy of exome sequencing to the proband. Screening of this mutation in family members showed that all patients were heterozygous and individuals with normal hearing were homozygous in the wild type, which indicated that p.M418K in *TMC1* was responsible for the hearing loss in this autosomal dominant family. *TMC1*, the transmembrane cochlear-expressed gene 1, was reported as the causative gene for both dominant and recessive hearing loss at the DFNA36 and DFNB7/11 [3]. Patients in this family (1304) showed post-lingual, bilateral, symmetric sensorineural hearing loss initially affected the mid and high frequency with mild level, followed by progression to severe or profound levels along with increasing ages, which was consistent with the phenotype of DFNA36 families reported previously [3], [14], [18], [19]. Alignments of the amino acid sequences of transmembrane channel-like gene 1 in different species showed that mutation in family 1304 was an orthologous to the p.M412K mutation reported in the hearing loss mouse model named Beethoven [5]. All these results strongly supported that the mutation of p.M418K in *TMC1* was associated with the hearing loss of patients in family 1304.

Notably, for the first time, our study reported a dominant mutation of *TMC1* in a large family from Chinese. During the past ten years, three dominant mutations in *TMC1* (p.D572N, p.D572H and p.G417R) from four DFNA36 hearing loss families have been reported [5], [14], [18], [19], i.e., the mutation of p.D572N and p.D572H were found in North American families [5], [18], [19]. The p.G417R mutation was found in an Iranian family [14].

*TMC1* is predicted to encode a transmembrane protein containing at least six membrane spanning domains, a cytoplasmic N- and C-termini, and a large cytoplasmic loop named TMC domain [24], [25]. The mutation found in this study, p.M418K, and the adjacent DFNA36 mutation of p.G417R, lie within a predicted second extracellular loop between the third and the fourth transmembrane domain (Figure 4E) [14], [24], while the amino acid, D572, is located in the region of TMC domain (Figure 4E) [24]. These dominant mutations must act via a gain-of-function or dominant-negative mechanism. The cluster of these dominant mutations of *TMC1* indicates that this region should be important for the proper function of the protein.

It's noteworthy that, for the first time, we have found a family in human with the mutation orthologous to p.M412K in *Tmc1* of *Bth* mouse model since it was found in 2002 [5]. The *Bth* mouse showed progressive loss of the Preyer reflex from around P30 with appeared normal structure of middle ear and inner ear[5], which was similar with the phenotype of late-onset and progressive hearing loss in family 1304. In-situ hybridization on mouse cochlear showed that *Tmc1* is expressed in both outer and inner hair cells from early stage of development [3], [5]. It may be required for cochlear hair-cell mechanotransduction as the integral components of the mechanotransduction complex [26]. Studies on the mutant mice that expressing the *Tmc1* ^Bth^ allele implicated that *Tmc1* was the component of the mechanotransduction channel in auditory hair cells of the inner ear [27]. And cells with the p.M412K point mutation in *Tmc1* reduced calcium permeability and single-channel currents [27]. Therefore, *TMC1* should act as a pore-forming subunit of the transduction channel and be involved in determining permeation properties [27].

To summarize, we found ABRs of the patients carrying the p.M418K mutation in *TMC1* showed a prolonged latency and abnormal waveform morphology for wave I to V, which was most likely a direct and causative link, although the mechanism was still obscure. The identification of the p.M418K in *TMC1* in family 1304 makes the *Bth* mouse an excellent animal model to study the mechanism for autosomal dominant hearing loss caused by *TMC1* mutation.

## Supporting Information

Figure S1 **The distribution of per-base sequencing depth in target regions for each sample.** Y-axis indicated the percentage of total target region under a given sequencing depth.
(TIF)

Figure S2 **Cumulative depth distribution in target regions for each sample.** X-axis denotes sequencing depth, and y-axis indicated the fraction of bases that achieves at or above a given sequencing depth. From the figure above, we can see about 75.50% of target region bases obtains at least 20x fold coverage, that is to say, about 75.50% of target region was covered by more than 20 reads. And about 89.10% of target region achieved at least 10x.
(TIF)

Table S1 **Primer sequences for p.M418 in exon16 and p.D572 in exon19.**
(DOCX)

Table S2 **Summary of effective data** **for exome sequencing.** * The region near target refers to flanking region within 200 bp of target regions. ** Total effective reads is the same meaning as the unique mapped reads which was stated in the pipeline above. Here the effective reads consist of two parts: i) the reads have only one best hit in the alignment. These reads comes from the unique region of genome ii) the reads have multiple best hits on the genome (the number of hits between 1 and 20), and they were randomly aligned onto the target regions. These reads mainly come from low complex genomic region, such as repetitive sequences, and account for about 4% of total effective reads. *** Target regions used here refer to genomic regions that the Exome array actually covered. The aggregate length of target is about 37.8 Mb.
(DOCX)

Table S3 **Summary of SNPs in Exome Sequencing for each Sample.** * Consensus genotype with quality score of at least 20. ** Intronic SNPs within 4 bp of exon/intron boundary. *** 5' UTR refers to 200 bp upstream of initiation codon, 3'UTR is defined as 200 bp downstream of termination codon.
(DOCX)

Table S4 **Summary of Indels in Exome Sequencing for each Sample.**
(DOCX)
