# Supplementary figures and images for: A Novel DFNA36 Mutation in TMC1 Orthologous to the Beethoven (Bth) Mouse Associated with Autosomal Dominant Hearing Loss in a Chinese Family
Source: PLoS One. 2014 May 14;9(5):e97064. doi: 10.1371/journal.pone.0097064 (PMC4020765; doi:10.1371/journal.pone.0097064)

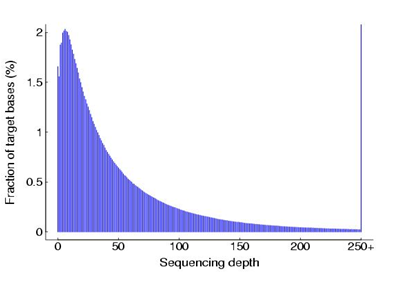

Supplement: Figure S1 — The distribution of per-base sequencing depth in target regions for each sample. Y-axis indicated the percentage of total target region under a given sequencing depth. (TIF) [file pone.0097064.s001.tif]

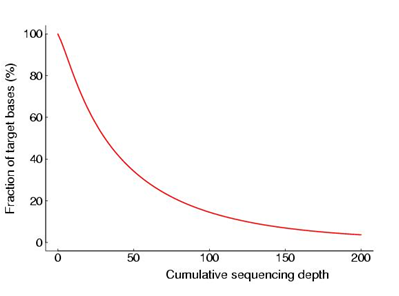

Supplement: Figure S2 — Cumulative depth distribution in target regions for each sample. X-axis denotes sequencing depth, and y-axis indicated the fraction of bases that achieves at or above a given sequencing depth. From the figure above, we can see about 75.50% of target region bases obtains at least 20x fold coverage, that is to say, about 75.50% of target region was covered by more than 20 reads. And about 89.10% of target region achieved at least 10x. (TIF) [file pone.0097064.s002.tif]
