# Supplementary material for: A Novel DFNA36 Mutation in TMC1 Orthologous to the Beethoven (Bth) Mouse Associated with Autosomal Dominant Hearing Loss in a Chinese Family
Source: PLoS One. 2014 May 14;9(5):e97064. doi: 10.1371/journal.pone.0097064 (PMC4020765; doi:10.1371/journal.pone.0097064)
Supplement: Table S1 — Primer sequences for p.M418 in exon16 and p.D572 in exon19. (DOCX) [file pone.0097064.s003.docx]

**Table S1 Primer sequences for p.M418 in exon16 and p.D572 in exon19**

| **EXON** | **Product length (bp)** | **GC%** | **Primer sequences (5’ to 3’)** | |
| --- | --- | --- | --- | --- |
| Exon16 | 835 | 37.3 | F | GTAAATTGCCACCTTCACT |
|  |  |  | R | AGAGCCAGCACACAGTCAAC |
| Exon19 | 373 | 41.8 | F | TATTGTTGCTGAAGGGAAGT |
|  |  |  | R | AGAGACACCTTTGATGACTG |
