# Supplementary material for: A Novel DFNA36 Mutation in TMC1 Orthologous to the Beethoven (Bth) Mouse Associated with Autosomal Dominant Hearing Loss in a Chinese Family
Source: PLoS One. 2014 May 14;9(5):e97064. doi: 10.1371/journal.pone.0097064 (PMC4020765; doi:10.1371/journal.pone.0097064)
Supplement: Table S2 — Summary of effective data for exome sequencing. * The region near target refers to flanking region within 200 bp of target regions. ** Total effective reads is the same meaning as the unique mapped reads which was stated in the pipeline above. Here the effective reads consist of two parts: i) the reads have only one best hit in the alignment. These reads comes from the unique region of genome ii) the reads have multiple best hits on the genome (the number of hits between 1 and 20), and they were randomly aligned onto the target regions. These reads mainly come from low complex genomic region, such as repetitive sequences, and account for about 4% of total effective reads. *** Target regions used here refer to genomic regions that the Exome array actually covered. The aggregate length of target is about 37.8 Mb. (DOCX) [file pone.0097064.s004.docx]

**Table S2 Summary of Effective Data for exome sequencing**

| **Exon Capture** | **V:6** |
| --- | --- |
| Initial bases on target: | 37563132 |
| *Initial bases near target: | 57118957 |
| Initial bases on or near target: | 94682089 |
| **Total effective reads: | 36248642 |
| Total effective yield(Mb): | 3179.56 |
| Average read length(bp): | 87.72 |
| Effective sequence on target(Mb): | 2017.25 |
| Effective sequence near target(Mb): | 363.91 |
| Effective sequence on or near target(Mb): | 2381.16 |
| Fraction of effective bases on target: | 63.40% |
| Fraction of effective bases on or near target: | 74.90% |
| **Average sequencing depth on target:** | **53.7** |
| Average sequencing depth near target: | 6.37 |
| Mismatch rate in target region: | 0.43% |
| Mismatch rate in all effective sequence: | 0.46% |
| Base covered on target: | 36940585 |
| **Coverage of target region:** | **98.30%** |
| Base covered near target: | 32698219 |
| Coverage of flanking region: | 57.20% |
| Fraction of target covered with at least 20X | 63.90% |
| Fraction of target covered with at least 10X | 81.00% |
| Fraction of target covered with at least 4X | 93.00% |
| Fraction of flanking region covered with at least 20X | 8.50% |
| Fraction of flanking region covered with at least 10X | 16.30% |
| Fraction of flanking region covered with at least 4X | 30.70% |

* The region near target refers to flanking region within 200bp of target regions.

** Total effective reads is the same meaning as the unique mapped reads which was stated in the pipeline above. Here the effective reads consist of two parts: i) the reads have only one best hit in the alignment. These reads comes from the unique region of genome ii) the reads have multiple best hits on the genome (the number of hits between 1 and 20), and they were randomly aligned onto the target regions. These reads mainly come from low complex genomic region, such as repetitive sequences, and account for about 4% of total effective reads.

*** Target regions used here refer to genomic regions that the Exome array actually covered. The aggregate length of target is about 37.8Mb.
