# Supplementary material for: A Novel DFNA36 Mutation in TMC1 Orthologous to the Beethoven (Bth) Mouse Associated with Autosomal Dominant Hearing Loss in a Chinese Family
Source: PLoS One. 2014 May 14;9(5):e97064. doi: 10.1371/journal.pone.0097064 (PMC4020765; doi:10.1371/journal.pone.0097064)
Supplement: Table S3 — Summary of SNPs in Exome Sequencing for each Sample. * Consensus genotype with quality score of at least 20. ** Intronic SNPs within 4 bp of exon/intron boundary. *** 5' UTR refers to 200 bp upstream of initiation codon, 3'UTR is defined as 200 bp downstream of termination codon. (DOCX) [file pone.0097064.s005.docx]

**Table S3 Summary of SNPs in Exome Sequencing for each Sample**

| **SNPs found in Exon Capture** | **V:6** |
| --- | --- |
| Number of genomic positions for calling SNPs: | 106194180 |
| Number of high-confidence genotypes*: | 69107503 |
| Number of high-confidence genotypes in TR: | 36253188 |
| Number of known SNP site in TR: | 154403 |
| Coverage of population SNPs in TR: | 142851 (92.52%) |
| Total number of SNPs: | 38130 |
| Synonymous-coding: | 8095 |
| Missense: | 6836 |
| Nonsense: | 57 |
| Readthrough: | 6 |
| Splice site**: | 325 |
| Intron: | 20139 |
| 5' UTRs***: | 1221 |
| 3' UTRs: | 1301 |
| Intergenic: | 150 |

* Consensus genotype with quality score of at least 20.

** Intronic SNPs within 4bp of exon/intron boundary.

*** 5' UTR refers to 200bp upstream of initiation codon, 3'UTR is defined as 200bp downstream of termination codon.
