# Supplementary material for: A Novel DFNA36 Mutation in TMC1 Orthologous to the Beethoven (Bth) Mouse Associated with Autosomal Dominant Hearing Loss in a Chinese Family
Source: PLoS One. 2014 May 14;9(5):e97064. doi: 10.1371/journal.pone.0097064 (PMC4020765; doi:10.1371/journal.pone.0097064)
Supplement: Table S4 — Summary of Indels in Exome Sequencing for each Sample. (DOCX) [file pone.0097064.s006.docx]

**Table S4 Summary of Indels in Exome Sequencing for each Sample**

| **Indels found in Exon Capture** | **V:6** |
| --- | --- |
| Total mumber of indels | 2220 |
| Ins-coding | 80 |
| Del-coding | 129 |
| Splice site | 44 |
| Intron | 1766 |
| 5' UTRs | 79 |
| 3' UTRs | 106 |
| Intergenic | 16 |
| Total insertion | 925 |
| Total deletion | 1295 |
| Heterozygous indels | 1283 |
| Homozygous indels | 937 |
